# Impact of Top Electrodes on the Nonvolatile Resistive Switching Properties of Citrus Thin Films

**DOI:** 10.3390/polym13050710

**Published:** 2021-02-26

**Authors:** Kai-Wen Lin, Ting-Yun Wang, Yu-Chi Chang

**Affiliations:** Department of Engineering Science, National Cheng Kung University, Tainan 701, Taiwan; kevlin.esc@gmail.com (K.-W.L.); n96094332@gs.ncku.edu.tw (T.-Y.W.)

**Keywords:** biodegradability, citrus, resistive memory

## Abstract

Natural citrus thin films on an indium tin oxide (ITO)/glass substrate were synthesized using the solution method for resistive random access memory (RRAM) applications. The results indicated that the citrus memory device possessed stable resistive switching behavior. For a clear understanding of the role of the interface reaction between the top metal electrode and the citrus film, we investigated the influences of various top electrode (TE) materials on the resistive switching in TE/citrus/ITO devices. In comparison with Au/citrus/ITO and Ti/citrus/ITO devices, the Al/citrus/ITO device can be reproduced with a DC voltage of more than 100 times while only showing a slight decrease in the ON/OFF ratio. In addition, the Al/citrus/ITO device exhibited a high ON/OFF ratio of over 10^4^ and an outstanding uniformity, which was attributed to the fast formation of a native oxide layer (AlO_x_), as confirmed by the line scan analysis. This indicated that the interface layer, created by the redox reaction between the Al electrode and citrus film, played an important role in the resistive switching properties of TE/citrus/ITO structures. These findings can serve as design guidelines for future bio-based RRAM devices.

## 1. Introduction

With the current rapid economic and technologic growth, the development of consumer goods has resulted in an increase the production of waste electric and electronic equipment. Therefore, electronics consisting of renewable and biodegradable materials are desirable to benefit our living environment [1,2,3,4].

The natural material citrus is one of the most popular fruit commodities in the world due to its nutritional values [5]. Citrus is biodegradable, which, in addition to its low toxicity, makes it an ideal candidate for the fabrication of environmentally friendly electronics. As the demand of data storage devices has grown at a prodigious rate, a simple fabrication process, non-volatility, and high-density integration have become basic requirements for modern memory [6,7]. Among various non-volatile memories, resistive random-access memory (RRAM), which is composed of a simple structure, has the superior characteristics of high-speed operation, low power consumption, and a long retention time [8].

For the advanced development of natural material-based RRAM devices, there is an important issue regarding how to optimize memory properties, such as the ON/OFF ratio, endurance, stability, and the dispersions of the resistive switching parameters, which require a clear understanding of the basic mechanism of resistance switching behavior. Currently, many reports have shown that resistive switching properties significantly depend on the metal electrode [9,10,11,12]. These reports have indicated that the contacts between the top electrode and insulator layer are related to resistive switching properties [9,10,11,12]. The interfacial reaction between the top electrode material and the natural material may cause either a favorable or an adverse influence on the resistive switching properties.

This work emphasized the relationships between the physical properties of citrus thin films and device characteristics. The simple-solution processed citrus RRAM could achieve an ON/OFF current ratio of over 10^4^. In addition, we also investigated how surface effects may change the electrical properties of citrus memory devices fabricated with various top electrodes. If we can understand the relations between the top electrodes and memory properties of citrus thin films, a stable and high device performance could more effectively be obtained. The memory properties and resistive switching mechanisms of the Al/citrus/indium tin oxide (ITO) RRAM devices were thoroughly analyzed. RRAM optimization via biomaterial engineering enables a deeper understanding of the material parameters that control the memory properties that are to be better explored for future bio-memory applications.

## 2. Materials and Methods

ITO-coated glass substrates were cut into 2.0 × 1.5 cm^2^ pieces and sequentially cleaned with acetone, methanol, and deionized (DI) water in an ultrasonic bath. A citrus solution was prepared using citrus powder mixed with DI water. The citrus concentrations were adjusted to 1.5, 1, and 0.5 mg/mL, which are denoted as C1.5, C1, and C0.5, respectively. The citrus solution was spun onto the cleaned ITO/glass substrates and baked at 80 °C for 15 min.

A citrus thin film was then fabricated by spin coating at 5000 rpm for 30 s. Finally, the Al electrodes with thicknesses of around 120 nm were deposited on top of the citrus thin film by radio frequency (RF)-magnetron sputtering, using an Ar working pressure of 20 mTorr and an RF power of 250 W. To investigate the electrical properties of C1 thin films with different metals, metal top electrodes (Au and Ti) were deposited by thermal evaporation through a shadow mask. The square-shaped top electrode area was 3 mm^2^.

Atomic force microscope (AFM) analysis (Dimension ICON with Nano Scope V controller, Bruker, Karlsruhe, Germany) was used to characterize the surface morphology and roughness of the samples. FTIR spectrometry was carried out using a Vertex 80v and Tensor 27 (Bruker) in the range of 4000−800 cm^−1^.

The electrical properties were characterized by an Agilent B1500 semiconductor parameter analyzer (Santa Clara, CA, USA). To gain further insight into the electrical characteristics, the devices were measured at temperatures ranging from 275 to 393 K to study the temperature dependence of the current characteristics. TEM analysis was carried out using a 200 kV JEM-2100F Electron Microscope (Jeol, Tokyo, Japan). XPS was carried out using a PHI 5000 Versa Probe (Kanagawa, Japan).

## 3. Results

**Figure 1:**
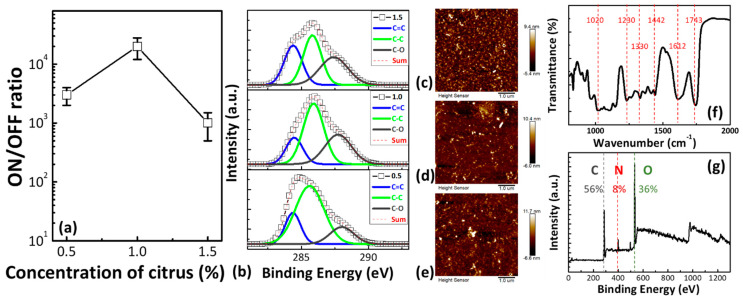
(**a**) The ON/OFF ratio as a function of the citrus concentration. (**b**) XPS C 1s spectra of the citrus thin film: C0.5 (bottom), C1 (middle), and C1.5 (top). Tapping mode 2D and 3D AFM topography images of (**c**) C0.5, (**d**) C1, and (**e**) C1.5 thin films. (**f**) FTIR analysis, and (**g**) XPS full spectrum analysis of the C1 thin film.

Figure 1a shows the ON/OFF ratio as a function of the citrus concentration. The ON/OFF ratio significantly decreased with increased citrus concentration up to 1.5%. The high resolution C 1 s spectra of the C0.5, C1, and C1.5 thin films at the three stages shown in Figure 1b were analyzed. The peaks at 284.5, 285.7, and 287.8 eV were attributed to the C=C bonds, *sp^3^* bulk bonded carbon C–C bonds, and *sp^3^* bulk bonded C=O bonds, respectively [13,14,15]. The C–C/C=C ratios were approximately 0.46, 0.53, and 0.4 for the C0.5, C1, and C1.5 thin films, respectively. After increasing the citrus concentration at 1%, the number of C–C *sp^3^* bonds increased, producing a low high-resistance state (HRS) current thin film [16,17]. The average ON/OFF ratio increased with the number of C–C *sp^3^* bonds.

The 2D and 3D AFM topography images of the C0.5, C1, and C1.5 thin films are shown in Figure 1c–e, respectively. The root-mean-square roughness (R_rms_) values of the C0.5, C1, and C1.5 thin films were around 1.52, 1.24, and 1.88 nm, respectively. A suitable citrus concentration enabled the formation of a smooth surface. A lower surface roughness may result in the better uniformity and higher yield of fabricated memory devices [18]. Changes in the citrus content helped tailor the number of functional groups and the surface roughness of the citrus thin films.

The FTIR spectra of gelatin are shown in Figure 1f. Significant peaks occurred at 1743 cm^−1^ due to the C=O stretching vibrations of carboxylic acid [19]. The absorption bands at 1612 were related to the –C(=O)–O stretching of the carboxylate groups and the C=O stretching vibrations of carboxylic acid [19].

**Figure 2:**
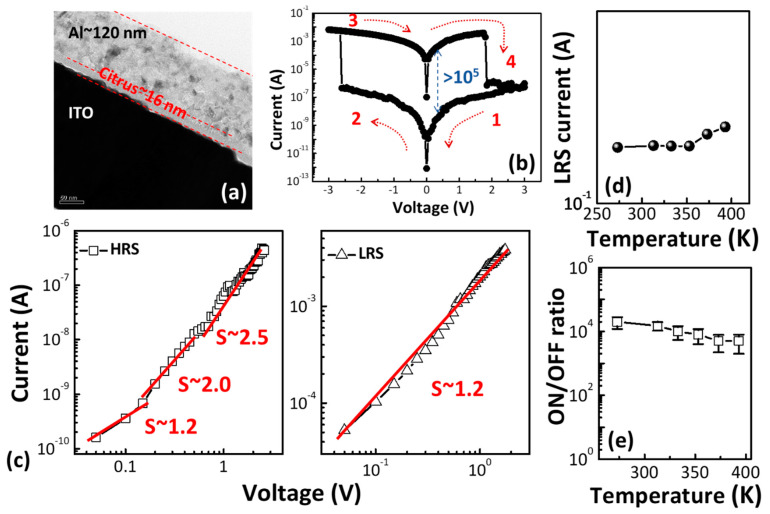
(**a**) TEM cross-section micrographs and (**b**) the typical current–voltage (*I–V*) characteristics of the Al/C1/indium tin oxide (ITO) structure. The bias sweep sequence is indicated by the arrows. (**c**) Double logarithmic plots of the high resistance state (HRS) (left) and low resistance state (LRS) (right) of the C1 memory device. (**d**) Temperature dependence of the LRS current of the C1 memory device. (**e**) The ON/OFF ratio as a function of the temperature from 273 to 393 K.

The peaks occurring at 1330 cm^−1^ were related to C–O–C stretching of the aryl–alkyl ether linkage [19]. The bands at around 1000–1100 cm^−1^ could be attributed to C–O–H alcohol bonds of saturated carbon, as well as to C–O stretching and C–O deformation [19,20]. Figure 2a shows the cross-sectional TEM image of the Al/C1/ITO structure. The XPS spectra of the C1 thin film reveal that the main element in the citrus thin film was carbon—the citrus thin film demonstrated a high carbon signal in the line scan profile (Figure 4d). The thicknesses of the Al and C1 thin films were around 120 and 16 nm, respectively. Figure 2b depicts the current–voltage (*I–V*) characteristic of the C1 memory device. Typical bipolar resistive switching was observed. When the applied voltage ranged from +3 to −3 V, the ON/OFF ratio of the C1 memory device was over 10^5^. Simultaneously, we plotted the *I–V* curve of the C1 memory device on a double log scale to investigate the conduction behaviors. In the negative bias region, there were three distinct regimes. The current at the HRS varied linearly with the voltage in the low-voltage region. Then, a slope of around 2 for 0.2 V < V < 0.5 V and a sharp current increased with a slope of over 2 for V > 0.5 V. This *I–V* curve exhibited the characteristics of the trap-controlled space-charge-limited conduction (SCLC) mechanism [21,22,23,24,25,26]. In the positive bias region, the C1 memory device showed a linear *I–V* relation, corresponding to the ohmic conduction mechanism. Based on the fitting result, the resistive switching behavior of the C1 memory device can be explained with the filament conduction mechanism [27,28,29].

To describe the possible conduction mechanism, the temperature dependencies of the low resistance state (LRS) current at 0.5 V are summarized in Figure 2d. The LRS currents of the C1 memory device increased with the increasing temperature between 273 and 393 K, indicating that the resistive switching material was semiconducting rather than metallic [30]. This suggests that the carbon-rich citrus thin film tended to form conducting filaments containing carbon elements.

The ON/OFF ratio as a function of temperature was measured to identify the stability at elevated temperatures of the C1 memory device, and the results are shown in Figure 2e. The ON/OFF ratio of the C1 memory device exhibited no significant effects over the temperature range of 273–393 K, indicating its excellent stability at elevated temperatures.

**Figure 3:**
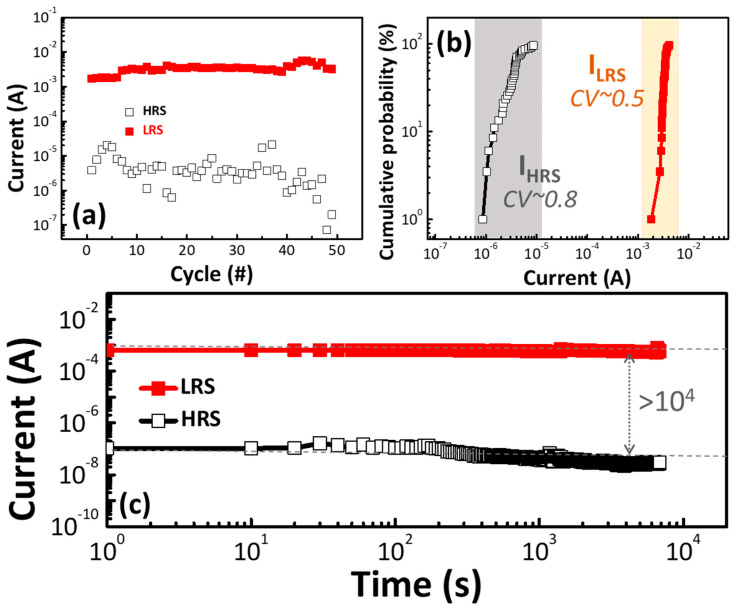
(**a**) Cycling I–V measurements of the C1 memory device. (**b**) The cumulative probability of the HRS and LRS current values for the C1 memory device under a read bias of 0.1 V. (**c**) Retention property of the C1 memory device at room temperature.

Figure 3a shows the I–V switching cycles of the C1 memory device. A switching of over 50 cycles was obtained, indicating that the resistive switching behavior is reproducible. The uniformities and stabilities of the C1 memory device were also measured. Figure 3b shows the statistical distribution parameters. The coefficient of variation (CV), defined as the ratio of standard deviation to the average value (AVG), was used to evaluate the distribution. Both the LRS and the HRS currents were measured at 0.1 V. The CV values of I_LRS_ and I_HRS_ were around 0.5 and 0.8, respectively. The distribution of the HRS was well-separated from that of the LRS, which provided a good margin of the ON and OFF states in the memory devices.

Figure 3c shows the retention ability of the C1 memory device, which was utilized to investigate the stability. The C1 memory device exhibited an ON/OFF ratio of over 10^4^, and no significant decay was observed within 104 s, indicating its excellent data retention capability. Good switching and device uniformity are important factors for bio-memory devices.

**Figure 4:**
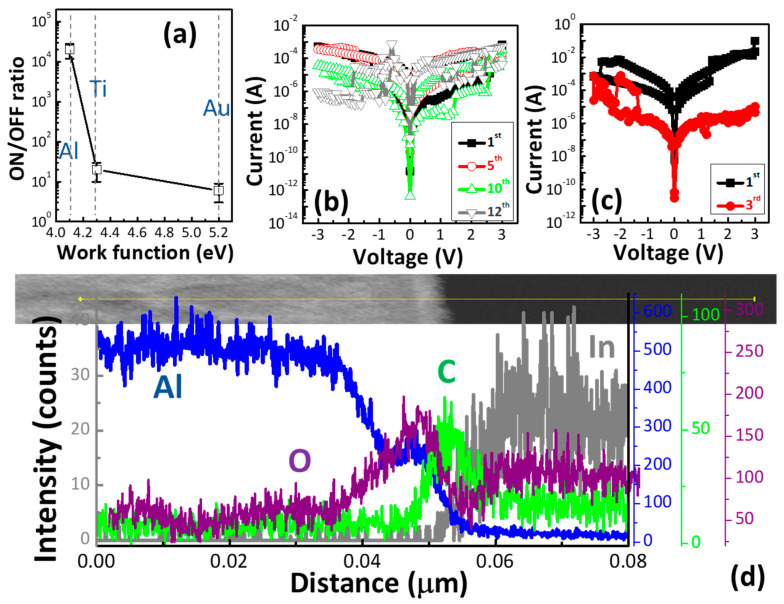
(**a**) The ON/OFF ratio of the C1 memory devices with various top electrodes. The switching cycles of the (**b**) Ti/C1/ITO and (**c**) Au/C1/ITO structures. (**d**) The line-scan profiles along the yellow line marked in the TEM image of the C1 memory device.

The metal/C1/ITO devices were demonstrated to further clarify the switching mechanism of the C1 memory devices with various top electrodes. Figure 4a shows the ON/OFF ratios of the C1 memory devices with the top electrodes of Al, Ti, and Au. Due to the lower work function leading to better resistive switching characteristics [9], using Al or Ti as the top electrodes showed better ON/OFF ratios compared with the Au/C1/ITO structure.

Despite the low work function (<4 eV) of Ti, the Al/C1/ITO structure exhibited a better ON/OFF ratio than the Ti/C1/ITO structure did. This result was attributed to the low HRS current due to the native AlO_x_ layer [31,32,33]. The low-HRS current favored the high ON/OFF ratio.

Figure 4b,c shows the *I–V* switching cycles of the Ti/C1/ITO and Au/C1/ITO structures, respectively. As shown in Figure 4c, the set process occurred in the positive region where the off-state changes to the on-state for bipolar resistive switching. The bipolar resistive switching behavior of the set and the reset voltages were in opposite bias in the Al or Ti/C1/ITO and Au/C1/ITO structure, which can be ascribed to the different work functions of top electrodes in the ITO.

Figure 4d exhibits the intensities for Al, C, O, and In elements as a function of distance. Al was also detected in the C1 thin film. The Al concentration throughout the entire C1 thin film decreased as the distance increased, indicating that Al diffused into the C1 thin film. The AlO_x_ layer between the Al electrode and the C1 thin film was detected. According to the line-scan profile of the C1 memory device, the AlO_x_ at the Al/C1 interface consisted mostly of Al and O. The formation of the AlO_x_ layer enabled the enhancement of the ON/OFF ratio of the C1 memory device. A good ON/OFF ratio is greatly needed for practical applications of resistive memory. These results thus support the strong role of the top electrode (TE) materials in dictating the switching and stability of resistive memory devices.

## 4. Conclusions

In summary, we investigated citrus thin films as a resistive layer in memory devices, as well as the influence of top electrodes on device performance. The citrus-based RRAM made with Al electrodes possessed an outstanding ON/OFF ratio of over 10^4^. An ON/OFF ratio of less than 10^1^ was found in Au/citrus/ITO RRAMs. In addition to the relatively high ON/OFF ratio, the Al/citrus/ITO RRAM also exhibited an acceptable uniformity, a stable switching cycle, and an outstanding stability at elevated temperatures compared with the Ti/citrus/ITO RRAMs. We attribute the better memory properties of the Al/citrus/ITO RRAM to the fast formation of a native oxide layer (AlO_x_), as confirmed by the line scan analysis. The bipolar resistive switching behavior of the set and reset voltages was in the opposite bias in the Al/citrus/ITO and Au/citrus/ITO RRAMs, which can be ascribed to the different work functions of the top electrodes in the ITO. The analysis of the temperature-dependent LRS current and fitting results indicated that the resistive switching behavior of the C1 memory device was related to the formation of filaments containing carbon elements. These findings allow for RRAM optimization via biomaterial engineering to be better explored in future bio-memory applications.

## Data Availability

We have no depository of publicly archived datasets analyzed or generated during the study. Data are available on request; contact please authors at their e-mail addresses.
